# Basic clinical equipment for physical health assessment in mid Essex inpatient units

**DOI:** 10.1192/bjo.2021.806

**Published:** 2021-06-18

**Authors:** Hesham Abdelkhalek

**Affiliations:** Essex Partnership University NHS Foundation Trust

## Abstract

**Aims:**

It is trust policy that the Basic Clinical Equipment for Physical Health Assessment should be available on each unit. The standard for this audit is therefore 100% completion.

**Background:**

This was a cross-sectional study of six mental health units across Mid Essex. We audited equipment and consumables in comparison to trust policies. For the purpose of the audit we designed an audit tool.

**Method:**

Overall compliance across all wards for all audited items was 77.5% (64.9% – 87.5%). Average compliance for equipment provision 83.3% (73.9 %– 91.3%) was greater than that for consumables 72.1% (58.8% – 82.4%).

**Result:**

When looking at the compliance on each unit separately, our data show that no unit has met the standard of 100% for equipment or consumables. From all units, one of the two older adults’ inpatient units had the highest overall compliance and highest compliance for consumables at 87.5% and 82.4% respectively while the perinatal unit had the lowest overall compliance and lowest compliance for consumables at 64.9% and 58.8 respectively. For the equipment compliance, intensive care unit and one of the older adults tied for the highest compliance at 91.3% while male inpatient unit and perinatal inpatient unit were tied the lowest compliance at 73.9%.

**Conclusion:**

This is an audit to assess the availability of Basic Clinical Equipment for Physical Health Assessment on inpatient units in Mid Essex. With an audit standard of 100% completion, it shows that overall compliance on all units was 77.5% which is not meeting our standard.

